# Core–shell nanoparticles used in drug delivery-microfluidics: a review

**DOI:** 10.1039/d0ra01032d

**Published:** 2020-05-13

**Authors:** Zahra Mahdavi, Hamed Rezvani, Mostafa Keshavarz Moraveji

**Affiliations:** Department of Chemical Engineering, Amirkabir University of Technology (Tehran Polytechnic) Tehran Iran moraveji@aut.ac.ir; Department of Petroleum Engineering, Amirkabir University of Technology (Tehran Polytechnic) Tehran Iran

## Abstract

Developments in the fields of lab-on-a-chip and microfluidic technology have benefited nanomaterial production processes due to fluid miniaturization. The ability to acquire, manage, create, and modify structures on a nanoscale is of great interest in scientific and technological fields. Recently, more attention has been paid to the production of core–shell nanomaterials because of their use in various fields, such as drug delivery. Heterostructured nanomaterials have more reliable performance than the individual core or shell materials. Nanoparticle synthesis is a complex process; therefore, various techniques exist for the production of different types of nanoparticles. Among these techniques, microfluidic methods are unique and reliable routes, which can be used to produce nanoparticles for drug delivery applications.

## Abstract 2

Developments in the fields of lab-on-a-chip and microfluidic technology have benefited nanomaterial production processes due to fluid miniaturization.

## Graphical abstract

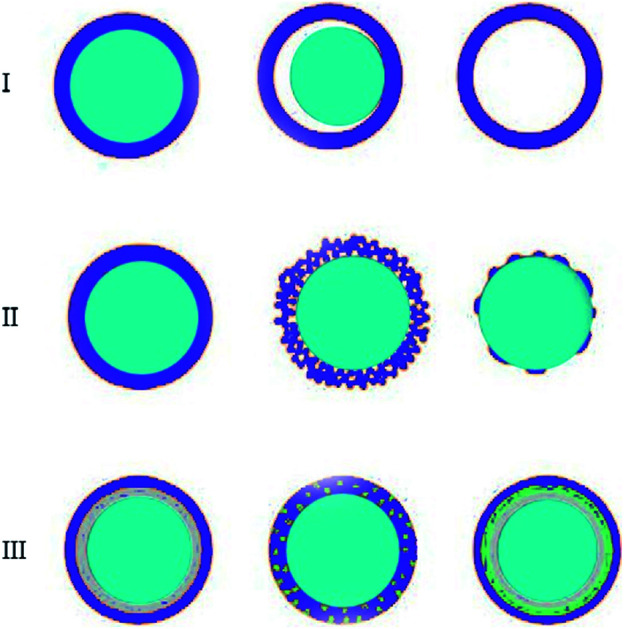

## 1 Introduction

**Fig. 1:**
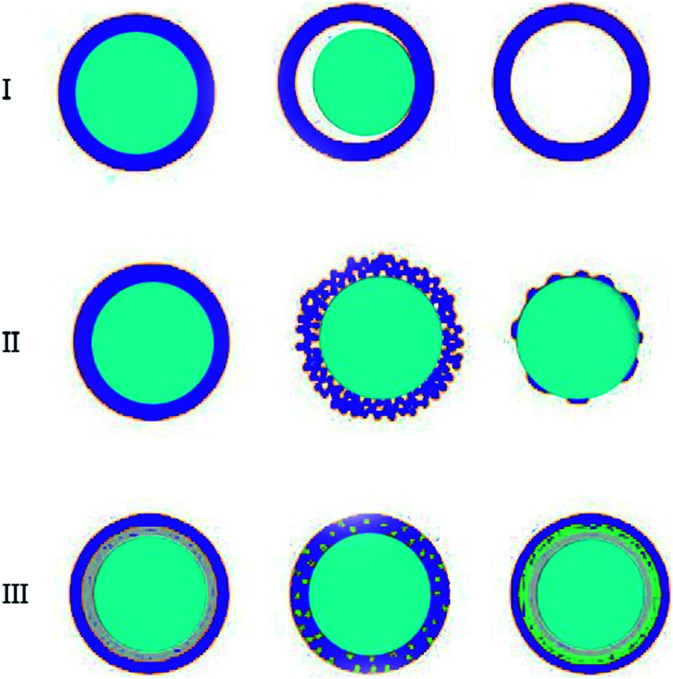
Core/shell particles scheme with different type of: (I) cores, (II) shells and (III) core/shell with complex structures.

Core–shell particles have become important over the past decade due to their potential in areas such as drug delivery, treatment with biomedical imaging, tumour therapy, and microfluidic devices.^1^ In the early studies, researchers examined nanoparticles because these particles had improved properties compared to the bulk materials. In the 1980s, researchers developed new particles, or unstable semiconductor particles, which had a higher efficiency than their allowed particles.^2,3^ In the 1990s, researchers developed new particles, or unstable semiconductor particles, which had a higher efficiency.^4^ However, the synthesis of nanoparticles is still a complex process requiring a wide range of techniques and the high demand for advanced materials has increased.^5^ The advancement of characterization techniques has helped in the creation of structures for these different core–shell nanocomposites.^6^ Nanomaterials that have different chemical and physical properties and overlap, play an important role for the complete understanding of some theoretical models, such as the classic particle-in-the-box model, for which classical physics laws have failed to provide an explanation. In addition, the combination of these materials at the nanoscale has led to the advancement of innovative technologies in many fields of science, resulting in the emergence of new fields for micro-strategies in the design and development of biological systems and devices (Fig. 1).^7,8^

### 1.1 Drug delivery

Drug delivery involves the release of a bioactive agent of an appropriate size at a desired rate. Cancer is a primary cause of death and despite the availability of various therapy modalities, cancer treatment remains a significant challenge.^9^ Chemotherapy is the primary therapeutic modality for cancer patients, and this has led to trials for a considerable number of chemotherapeutic anticancer drugs. Nevertheless, the main limitation to the clinical use of drugs is their broad bio-distribution and quick half-life. The significant disadvantage of traditional drug delivery methods is their weak selectivity; consequently, healthy cells are exposed to the cytotoxic effects of drugs. In most cases, an inadequate portion of the applied drug arrives at the tumour position.^10^ This weakness is addressed by a high drug dosage, which adds to their unwanted side effects. Accordingly, it is necessary to promote new drug delivery systems to overcome these weaknesses and to increase the effectiveness of cancer therapies.^11^ These new methods deliver the drug to the tumour position and decrease their side effects. Various types of drug delivery methods are developed for various healing applications. Nanoparticles (NPs) are one of the most commonly applied carriers that have great interest for their potential in drug delivery due to their ability to arrive at the therapeutic objectives at appropriate times and doses.^12^

### 1.2 Microfluidic devices

The double emulsion method is an extensively utilized route for forming core–shell nanoparticles.^13^ The synthesis process involves the evaporation of the solvent, emulsion, purification, and sonication of the created NPs, in addition to other multi-step and complex processes. Further, the synthesized NPs have moderate drug recovery rates, weak size distributions, and complex nanostructures. Due to difficult fluid management in bulk, microfluidic methods are utilized to control the formation and the size of the particle (Table 1).^14^

**Table 1:** The advantages and challenges of the microfluidic system.^15^

Advantages	Challenges
Large proportion to volume	Many substrates of the device have poor solvent-resistance and are not resistant to high temperatures
Less sample consumption	Sensitive to channel blocking, which may also change the mix
Effective manipulation of reagents	Special substrates (such as glass or silica) or molds (Su-8 based) are expensive and include clean room features
Adequate mixing, controlled residence time and proper reaction conditions	Limited in line description techniques
The kinetic study is well-controlled due to heat and mass transmission	Hardening in the line of refining and extraction
Synthesis with high efficiency	Prevention and post-operative work are not fully automated
Nanoparticles with adjustable size and smaller size distribution, which results in increased physical/chemical performance	
Simple synthesis potential with multi-stage integration in a single chip	
Potential for line identification, optimization and feedback control	

Microfluidic techniques are innovative and well-defined methods that use a minimal quantity of reagents, affording the specific management of physical processes and mixing at the microscale. To synthesize several nanostructures, such as polymeric systems, homogenous nanoparticles, and liposome–hydrogel particles, the continuous flow microfluidic approach is an ideal platform. In particular, microfluidics are a useful bottom-up method for synthesizing nanoparticles with significant control over the morphology, composition, and size distribution.^16^

## 2 Core/shell nanoparticle classification

**Fig. 2:**
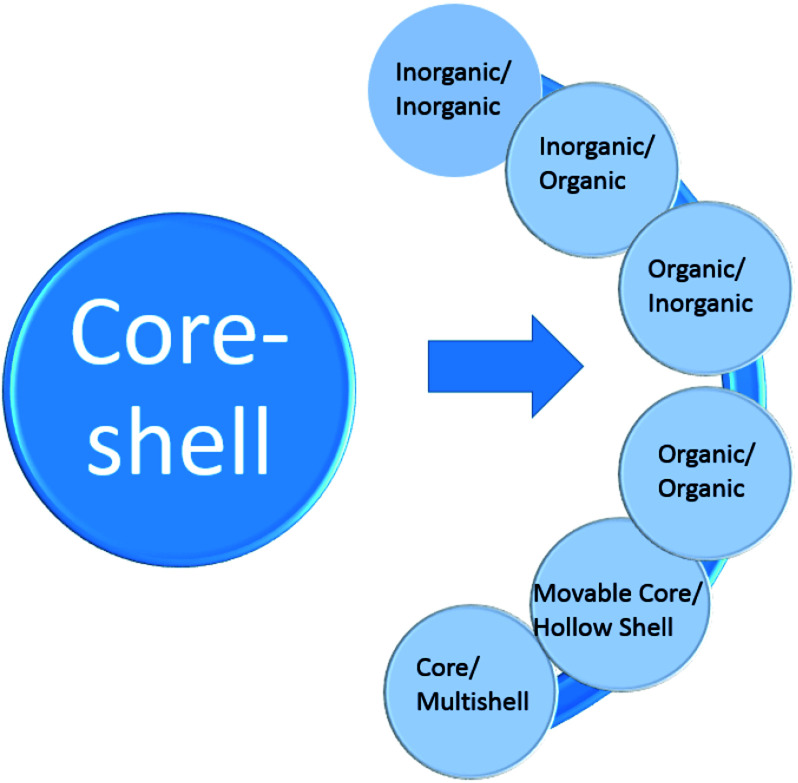
General core–shell classification.

**Fig. 3:**
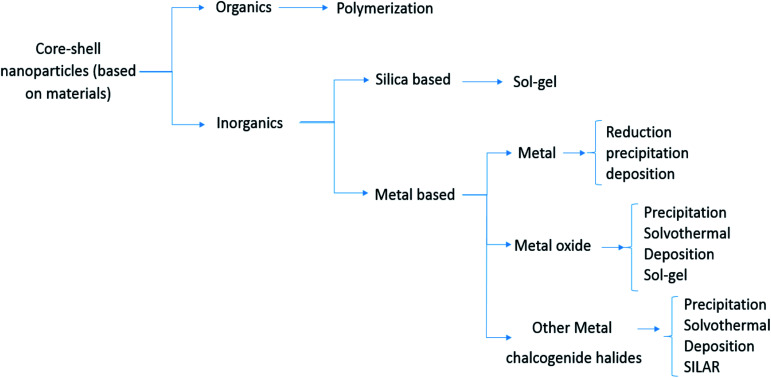
Classification of CSs based on materials.

In general, the most important features of CSNs as a building block model for functional materials^17^ are solubility,^18^ reactivation involving optical stimuli,^19^ narrow size distribution,^20^ stability,^21^ core and shell processing,^22^ and autonomic capability.^23^ Many types of nanoparticles can be classified as different types of core–shell materials, such as: single or multiple materials in plane, and core/shell or composite nanoparticles. The core/shell and composite particles are composed of two or more substances, while simple nanoparticles are made of a single material.^24^ Generally, the definition of core/shell nanoparticles relies on possessing an internal matter and an outer layer material. Different types of core–shell materials can combine in a range of different compositions, as shown in Fig. 2 and 3. The selection of the core–shell material primarily depends on the objective.^25^ There have been outstanding results reported for core–shell materials used in drug delivery (with their respective advantages and disadvantages of them), as presented in Table 2.

**Table 2:** Important research in the field of core–shell materials for drug delivery

Advantages	Disadvantages	Reference
Investigating the parameters affecting the nanoparticles of the core–shell structure	Failure to pay attention to the aspects of nanoparticle stability	Chaudhuri^130^
Advancement in functional core–shell nanoparticles of polymers in medical biotechnology	Single dimensional polymer nanoparticle analysis	Pradip Paik^131^
Fluorescent core–shell silica nanoparticles toward lab on a particle for nanobiotechnology and silica nanoparticles reduce cost in comparison with gold	Lack of comprehensive development in many areas of treatment	Andrew Burns^132^
Effect of particle size on the exchange bias field for core–shell structured nanoparticles	Lack of comparison and review with other materials and methods	Abdullah Ceylan^133^
Attempted to present and provide biomedical applications of all core–shell nanoparticles	Multidimensional investigation and no review of alternative solutions	Krishnendu Chatterjee^134^
Elaborate core–shell nanoparticles as biocompatible carrier for oral administration	Failure to compare the sustainability of the method and the usefulness of the method	Linguyn Chen^135^
Core–shell structured hollow mesoporous nano capsules as potential platforms for simultaneous anticancer drug delivery and cell imaging	Unilateral review and poor evaluation of progress in recovery and economic efficiency	Yu chen^9^
Synthesis and characterization of magnetic properties of core–shell structured Fe/Au nanoparticles	Failure to compare with previous methods and the usefulness of this method	Sung jin cho^136^
Core–shell structured nanoparticle layered nanochip provides convenient and easy-to-operate cancer diagnosis	Lack of comparison with previous methods and expensive gold use in academic research	Tatsuru Endo^137^
Synthesis of magnetic upconversion luminescent and mesoporous silica core–shell structured nanocomposites as drug carriers	Minimal comparison of the extent to which side effects and the previous methods are effective	Shili Gai^138^
Protein release kinetics for core–shell hybrid nanoparticles *via* the layer-by-layer self-assembly technique for delivery of bio macromolecules and possesses a high loading capacity for the encapsulation	Lack of comparison with previous methods and no indication of the usefulness of this method	Ziyad Haidar^139^
Core–shell fluorescent magnetic silica coated composite nanoparticles for bioconjugation and this method is easily manipulated	Not expressing the use of this method and its advantage in other ways	Rong He^140^
Synthesis and characterization of chitosan polyacrylicacid nanoparticles	No statement utility of method review and comparison with the previous methods	Yong Hu^141^
Used monodispersed core–shell spherical colloids with movable cores for microencapsulation	Failure to state the progress with previous methods and the amount of economic saving compared to other methods	Kaori Kamata^142^
Large scale synthesis of Ni–Ag core–shell nanoparticles with magnetic, optical and improved anti-oxidation properties as compared to Ni nanoparticles	Not expressing the amount of profitability and comparing aforementioned method with other methods	Chung Che Lee^143^
Sequentially releasing dual drug-loaded PLGA-casein core–shell nano medicine and significant improvement in the plasma concentration, residence time and circulation half-life of nano encapsulated to bare drug	Lack of comparison with other methods and did not express the amount of benefit	Sreeja Narayanan^53^
Triple functional core–shell structured upconversion luminescent nanoparticles covalently grafted with photosensitizer for magnetic resonance imaging and photodynamic therapy *in vitro* possessing unique advantages with more convenience	Complexity of the method and not comparing with recent methods	Xiao-Fei Qiao^144^
Gold/iron oxide core hollow shell nanoparticles have potential as magnetic resonance imaging agents	Failure to compare with previous methods and the degree of effectiveness of this method	Elena Shevchenko^145^
Design and application of core–shell and nano rattle multifunctional uniform nanostructures in commercial scale quantity	Unilateral and investigation not compared with previous methods	Ahmed Toni^146^
Assessment of the sensitivity of core–shell parameters derived using the single particle soot photometer to density and refractive index	Not expressing the advantage of the method in comparison to other methods and economic cost	J. W. Taylor^147^
Optimal nano-in-nano vector, including a drug nanocrystal core and a polymer shell, is successfully invented by a microfluidic glass capillary device	—	Liu^148^
Developed a three-stage microfluidic device for delivering a hydrophilic drug	—	Zhang^149^
Produced chitosan microcapsules with acid-triggered burst release property by a microfluidic device	—	Liu^150^
Created new PSi-based nanovaccines for cancer immunotherapy with nanoprecipitation in glass-capillary microfluidics device	—	Fontana^151^
Developed an excellent one-step microfluidic self-assembly method to create an advanced nanocomposite by encapsulating a PSi nanoparticle	—	Liu^152^
A hybrid nanocomposite covered with porous silicon nanoparticles and stimuli-responsive material utilized to load chemotherapeutics	—	Liu^153^
Created a microgel as an *in vitro* cell culture platform to manage injectable tissue constructs using the microfluidic device and photopolymerization process	—	Cha^154^

### 2.1 Core–shell nanoparticles for drug delivery in microfluidics

Biodegradable nanoparticles are utilized for drug delivery because of their enhanced healing and reduced side effects. Further, various traditional techniques can improve the lower drug loading efficacy.^26^ The microfluidic method provides the conditions to synthesize nanoparticles with a narrow size distribution, enhanced reproducibility, excellent encapsulation efficiency, and real-time analysis of the synthetic process. To take advantage of these features, researchers synthesized drug-loaded nanoparticles with a flow force microfluidic device.^27^ Nanoparticles can be made by the precipitation of polymer from an organic solvent to a non-solvent inside a hydrodynamic flow focusing microfluidic device. This microfluidic synthesis method accelerates the manipulation of nanoparticles for treatment,^28^ and is utilized by several researchers to synthesize many nanoparticles for drug delivery.

### 2.2 Mesoporous silica-based core–shell nanoparticles

In the past decade, mesoporous silica-based nanoparticles (MSNs) with properties such as vast surface area, uniform and high pore volume, adequate pore diameter, and superior biocompatibility, have been used in drug delivery and biomedical applications. As a result, the number of drug delivery devices based on MSNs has increased to take advantage of the conditions under which the devices can be active, like light, competitive binding, pH, temperature, and redox activation. These devices have exhibited the most ideal behaviour in terms of the sustained drug release.^29^

Several attempts have been made to improve the structure design and effective optimization to increase the rate of development of MSNs-based drug release devices. Several organic and inorganic materials, like magnetic components, gold nanoparticles, and polymers, can be combined with MSNs to develop effective hybrid MSNs for controllable drug delivery, which can be classified as structured MSNs and hollow/rattle-type MSNs.^30,31^

#### 2.2.1 Core–shell structured MSNs

Several practical nanomaterials with a diameter of 10 nm can be used by MSNs to provide additional desirable characteristics and result in core–shell structured MSNs. Commonly, an organic solvent, including the up-converting nanoparticle suspension, is blended to a specific surfactant solution. Next, a silica precursor is fused in the blended solution in order to obtain silica condensation. As a result, preceding the mesoporous silica synthesis, the nanomaterials can be covered with a standard silica layer from the silica precursors. Later, another specific molecule is added to create a mesoporous silica coating (Table 3).^43^

**Table 3:** Some mesoporous silica-based nanoparticles used in drug delivery

Coated material	Main structure	Reference
Au	MSN	32
AuNCs	MSN	33
AuNRs	MSN	34
Pd–Ag	MSN	35
Fe_3_O_4_	MSN	36
Fe_2_O_3_	MSN	30
UCNPs–SiO_2_	MSN	37
CaWO_4_:Tb^3+^	MSN	38
PDA/LA	MSN	39
PDA–MoSe_2_-wrapped doxorubicin (DOX)	MSN	40
PEG	MSN	41
PEI	MSN	42

This method has been used to combine various particles with mesoporous silica. For instance, iron oxide, gold, and polymers, which can contribute additional fascinating functionalities to MSNs, such as fluorescence, have been added into mesoporous silica to create beneficial core–shell structured MSNs.^36^

Core–shell structured MSNs have been created to transport drugs toward target cells with several internal and external triggers to achieve controllable release behaviour. For example, multi-practical core–shell structured MSNs, which are a mixture of MSNs with the drug-loading capability and metal particles with plasmonic features, have been created with indirect photothermal triggers to control drug release.^32^

#### 2.2.2 Hollow/rattle-type MSNs

Hollow/rattle-type MSNs can realize a significant drug-loading capacity because of their hollow internal structure, which increases the amount of fillable space. This special drug-loading capacity plays an essential role in improved chemotherapy and cancer therapy. As a result, numerous reports have described novel synthesis strategies for hollow/rattle-type MSNs.^38^

Hollow/rattle-type structured practical MSNs have large empty spaces and possess a more significant drug-loading capacity in comparison to single MSNs. The core and shell can be easily modified by several organic groups to facilitate both targeted drug delivery and drug loading. Furthermore, the mesoporous silica shell can be used to preserve the core to postpone core decomposition and prevent early drug release, and achieve the required drug delivery at the targeted positions by enabling an appropriate release control mechanism. Hence, numerous drug delivery systems utilizing hollow/rattle-type MSNs have been invented in order to encapsulate drug molecules.^44^

### 2.3 Organic polymeric core–shell nanoparticles

Core–shell polymeric particles have been studied for drug delivery applications and a series of practical techniques have been created; a well-known and widely used technique is the encapsulated structure approach. Core–shell polymeric particles can be created by emulsion, coaxial devices, and other techniques in order to encapsulate the drug.

The encapsulated structure method primarily involves an insoluble lipophilic layer on the surface that acts as a drug barrier to contain the drug in its internal hydrophilic conditions and prevent drug release until it is in the correct release position, at which point the barrier bursts due to the conformation changes. As a result, the side effects are significantly reduced and extravasation to tumour sites is notably improved.^45,46^

### 2.4 Metal and metal oxide core–shell nanoparticles

Magnetic materials such as iron and useful metals such as silver and gold are two typical kinds of metal and metal oxide nanoparticles that have used as drug delivery tools. The magnetic nanoparticles are mainly utilized as a core because their properties are unaffected by shell layers; however, rarely used as a shell like Au for its effective operational surface.^47^ Various inorganic nanomaterials are unsuitable and toxic to the human body. However, MnO,^48^ TiO_2_,^47^ and ZnO nanoparticles have been considered in drug delivery.

Magnetic nanoparticles like Fe are fascinating for controlled drug treatment purposes due to their biocompatibility and paramagnetic behaviour, which help the drugs flow to target cells by utilizing a magnetic field to control the route. The mentioned magnetic NPs have been examined as drug carriers in cultivated cell groups and *in vitro* methods in order to simulate the physiological systems of the human body. However, Fe NPs are toxic because of their oxidizability and free radical release into the blood flow.^70,71^ On the other hand, the stabilization of Fe NPs can be achieved by covering them with a metal shell and modifying the surface chemistry within the carrier and the drug. For example, a gold covering exhibits excellent adsorption for amines in anticancer drugs, and decreases the aggregation of particles by steric interference (Table 4).^72^

**Table 4:** Some polymer core–shell nanoparticles used in drug delivery

Core	Shell	Refrence
Oxidized sodium alginate	Chitosan	49
BSA	PLGA	50
PLGA-PEG-PLGA	Lipid	51
PLGA	DLPC	52
PLGA	Casein	53
Dextran	PLGA-PLA	54
PLLA	PLGA	55
Pectin	Alginate	56
PLLA	PLGA	57
Aqueous solution	Lipid	58
Chitosan	Cholesterol	59
PLGA	Alginate	60
PLGA	PDLLA	61
PEG	PCL	62
Cholesterol	Chitosan	63
PLGA	PEG	45
Polystyrene	Polybutyl-2-cyanoacrylate	64
Polycaprolactone	Dextran	65
PLGF-PLAF	PLEO	66
*N*-Isopropylmethacrylamide	*N*,*N*′-Methylene	67
Polystyrene	Polybutyl-2-cyanoacrylate	64
Ferrite impregnated acrylonitrile	Acrylamide	68
PMMA	PEI	69

Generally, metal NP utilization is a cost-effective method in drug delivery especially due to the simplicity, drug-vehicle nanoconjugate stability, and significant effectiveness.^73^ Nevertheless, metal NPs are toxic to the human body, and thus sensitive handling is needed for various sensitive tissues. Moreover, controlling the drug targeting process externally by magnet for distant tissues is challenging. Lastly, hydrophilic drugs cannot continuously travel in the human body as uncovered molecules without alerting a hydrophilic molecule, which triggers undesired interactions.^74^

Because of their specific physical properties and ability to operate at the molecular level of biological interactions, MNPs can be coated by various materials such as polymers,^75^ silane,^76^ silicon oxide,^77^*etc.*

#### 2.4.1 MNPs coating with polymers

The typical way to address nanoparticle oxidation stability in drug delivery usage is to coat the MNPs with polymers. Hence, the polymeric shell carries the drug and releases the coated drug upon its biodegradation.^75^

#### 2.4.2 MNPs coating with silane

Silanes are available through various amine groups and can improve the magnetite nanoparticle surface functionality for protein conjugation. Hence, MNPs coated with silane results in high-quality materials for the magnetic drug delivery system.^76^

#### 2.4.3 MNPs coating with SiO2

Superparamagnetic nanoparticles coated with a silicon oxide layer were created through standard microemulsion technology and then were used to encapsulate drugs, which can offer advantages over traditional drug delivery devices through improved dosing accuracy, better performance efficiency, and higher compliance.^77^

## 3 Microfluidics systems used in core–shell drug delivery systems.

### 3.1 Microfluidic approaches for making single and double emulsions

Droplets, which are produced by microfluidic devices in comparison to continuous-flow methods, can simultaneously control the quantity, composition, diffusion and limited cross-contamination, which are excellent terms for making complex particles for drug delivery approaches.^78^ Various microfluidic methods have been developed and used for creating single droplets and double or multiple emulsions. These methods are divided into two main classes: parallel and droplet based (Table 5).^79^

**Table 5:** Some metal and metal oxide core–shell nanoparticles used in drug delivery

Core	Shell	Reference
Fe	Au	70
Ag	Poly(*N*-isopropylacrylamide-co-acrylic acid)	80
Silver	ZnO	47
Gold	ZnO	47
Au@Ag nanorod	ZIF-8	81
ZnO–DOX	ZIF-8	82
Ag	TiO2	83
SiO_2_	Au	84
Au	PEG	85
Fe	*N*-Isopoly-acrylamide	71
Au	PEG-amino acid	86
Ag@SiO_2_	mTiO_2_	87
Ag	SiO_2_	88
Fe_3_O_4_	PMAA	89
Fe_3_O_4_	Au	90
Fe_3_O_4_	Chitosan	91
CaCO_3_@Fe_3_O_4_	PMMA	92
MnO	ssDNA	48
Au-oleic acid	*N*-Isoproprylacrylamide	93

Continuous and parallel flow-based microfluidic devices are utilized for creating self-assembled nanoparticles as a drug delivery system.^94^ Many studies have been performed on microfluidic multiphase flows. The essentials and physical parameters, such as flow regimes, dimensionless numbers, and interfacial effects, in multiphase flow are widely studied, which are summarized below.

**Fig. 4:**
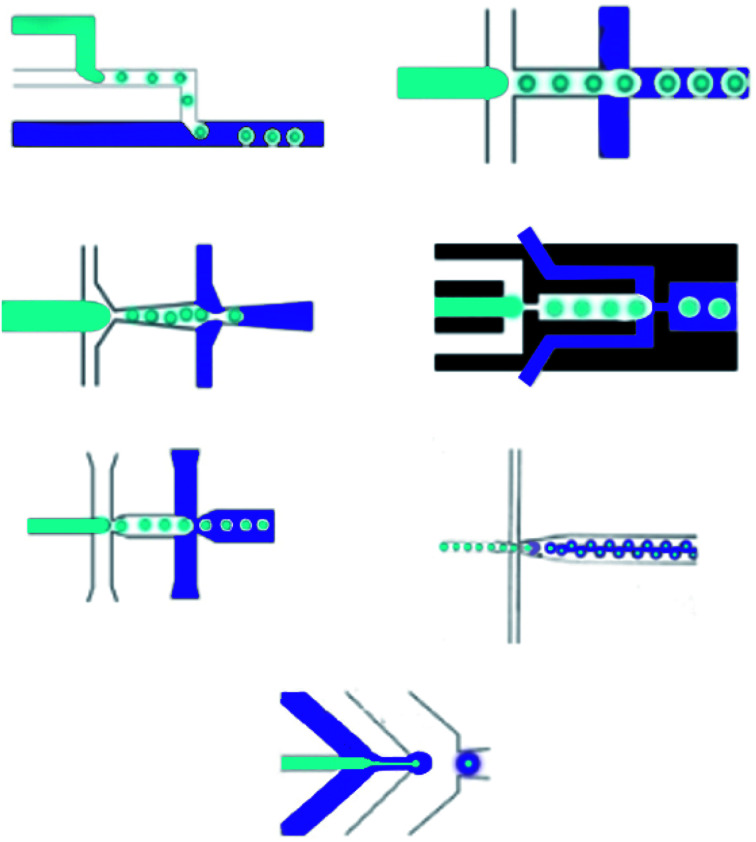
Two-step method microfluidic devices for making double emulsions.

The remarkable advantage of the droplet, which is made by microfluidics, is the ability to create monodisperse droplets with a size distribution lower than 1% size deviation. Besides, droplet sizes can be perfectly controlled with passive methods by tuning the microfluidic device geometry, interfacial tension and viscosity of liquid phases, flow rates, and pressure, or with active methods by electrical forces, magnetic force, temperature, and acoustic force.^78^ Generally, microfluidic methods for manipulating single emulsions include the dispersed phase injection into the continuous phase, which is immiscible or partly immiscible liquid, into a unique microfluidic device;^95^ droplets suddenly become separated at the junction where the phases meet. Among microfluidic systems the T-junction, flow-focusing and co-flowing microfluidic devices mostly utilize. The T-junction pattern is a horizontal straight channel with two branches.^96^ In the flow-focusing shape, the flow-focusing segment structures are different, but the droplet structure mechanisms are the same. The dispersed phase is pumped into a horizontal channel, and the continuous phase flows into side channels.^97^ When the two phases meet, the inner fluid breaks into spherical droplets. In the flow-focusing scheme, a shearing force is applied by the continuous phase from the side channels, providing higher stability and a controllable environment to produce droplets.^98^ Compared to 2D devices, which are made in various substrates such as silicon, PDMS, and glass, microfluidic devices with 3D channel formations are used to manage droplet structure. Besides, 3D devices that can be manipulated by capillary channels are desirable because they can entirely restrict a dispersed phase into a continuous phase.^99^ Capillary devices are made of a glass capillary cylinder inserted into another capillary. Immiscible liquids coming from the capillaries made by co-flowing streams can create monodisperse droplets. Another microfluidic device for creating droplets contains microchannels and layer emulsification.^100^ In two-phase microfluidic systems,^101^ many dimensionless numbers are essential functions, containing Re, Ca, We and the flow rate ratio (*φ*), which are introduced below (Fig. 4).

The Reynolds number represents the relative ratio of inertial forces to viscous forces;^102^ the capillary number expresses the relative effect of viscous forces on interfacial tension forces;^103^ the Weber number introduces the effect of inertial effects on the surface tension;^104^ the flow rate ratio (*φ*) represents the flow rate ratio of the continuous phase to the dispersed phase.^105^ Because of the micro-scale in microfluidics, Re is extremely small (smaller than 1), making We and Ca more impactful than Re; hence, the interfacial effects govern the system. The wettability of the microfluidic device channel walls are significant in selecting the emulsion types that can be created, and setting the flow patterns that can be made. In general, water-in-oil droplets (W/O) are created in hydrophobic systems;^106^ however, oil-in-water emulsions (O/W) are created in hydrophilic systems.^13^ Moreover, dictated flow patterns are made when the continuous phase effectively wets the channels. However, the incomplete wetting process creates disorganized flow patterns. In T-junction devices, dripping, squeezing, and jetting regimes, which are important in microfluidics, are numerically studied. Likewise, in co-flowing and flow-focusing microfluidic devices, three regimes are set to contain jetting, tip streaming, and dripping.^107^

### 3.2 Core–shell as double emulsions

Double emulsions are made of two droplets inside each other. Due to the controlled internal formations and the controlled sizes of droplets, the manipulation of multiple emulsions is extremely difficult and complicated. Fortunately, one of the advantages of microfluidics is that it is possible to make multiple emulsions with a requested droplet size, composition, and structure, which is beneficial for creating microcapsules, core–shell microspheres, microparticles, multi-cored microcapsules, effective colloidosomes with shells, and extremely complex materials.^108^ Monodisperse double emulsions are made by various droplet-based microfluidic systems, which are categorized in two classes: one-step and two-step methods.^109^

#### 3.2.1 Two-step methods

**Fig. 5:**
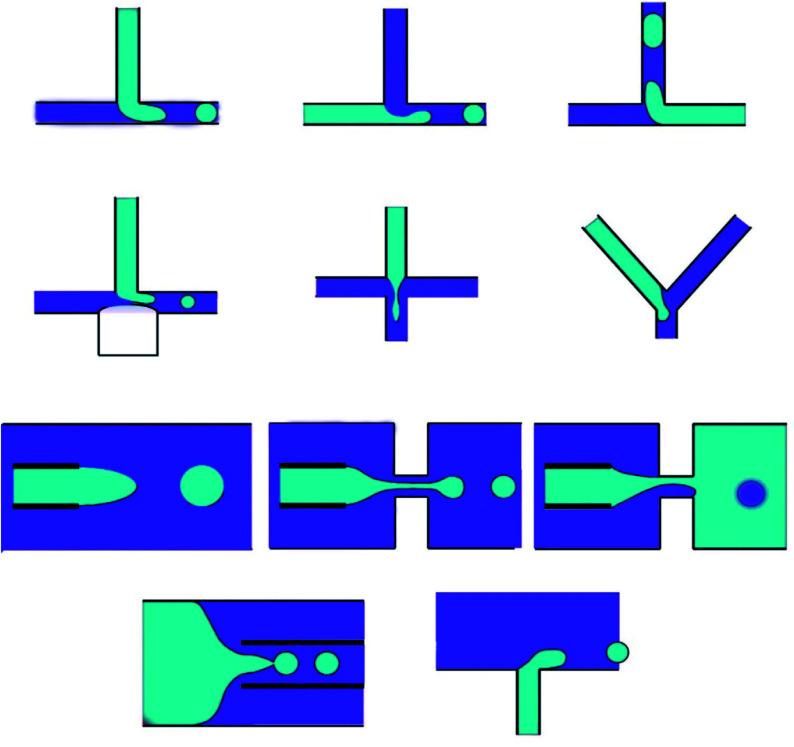
One-step methods microfluidic devices for making double emulsions.

Double emulsions are usually made by a two-step method, which at the beginning makes the inner droplets, then encapsulates it in a second emulsification step. Two-stage microfluidic devices are extensively utilized for creating W/O/W and O/W/O double emulsions.^110^ The two-step approach applies various combinations of primary structures, such as co-flowing, T-junction, cross-flowing, flow-focusing, and cross-flowing with opposite wettability, that are serially connected and create double emulsions by making the inner droplet in the first structure and the outer droplet in the second structure, which can contain two flow-focusing,^111^ two co-flowing, one T-junction and one flow-focusing, *etc.* Additionally, moving-wall geometries are merged with microfluidic devices for controlling the single and double emulsion size. The inner and outer droplet sizes, the number of inner droplets, and the thickness of the middle layer are set by setting the microfluidic structure and the flow rates.^112^ Furthermore, managing the wettability in the two-step approaches is significant. Hydrophilic channels require changing to hydrophobic, for example, when utilizing a silane coupling approach; however, hydrophobic PDMS, which is a common material for creating a microfluidic device, must be made partly hydrophilic, which is difficult due to the inert nature of the PDMS. Surface oxidation with oxygen plasma is a standard method to modify PDMS; however, the exposure to oxygen plasma cannot create long-term stable surfaces. The other way of preventing spatial-surface modification is to select the appropriate solvents precisely; therefore, double emulsions can be created without surface treatment in PDMS microfluidic devices (Fig. 5).^113,114^

**Fig. 6:**
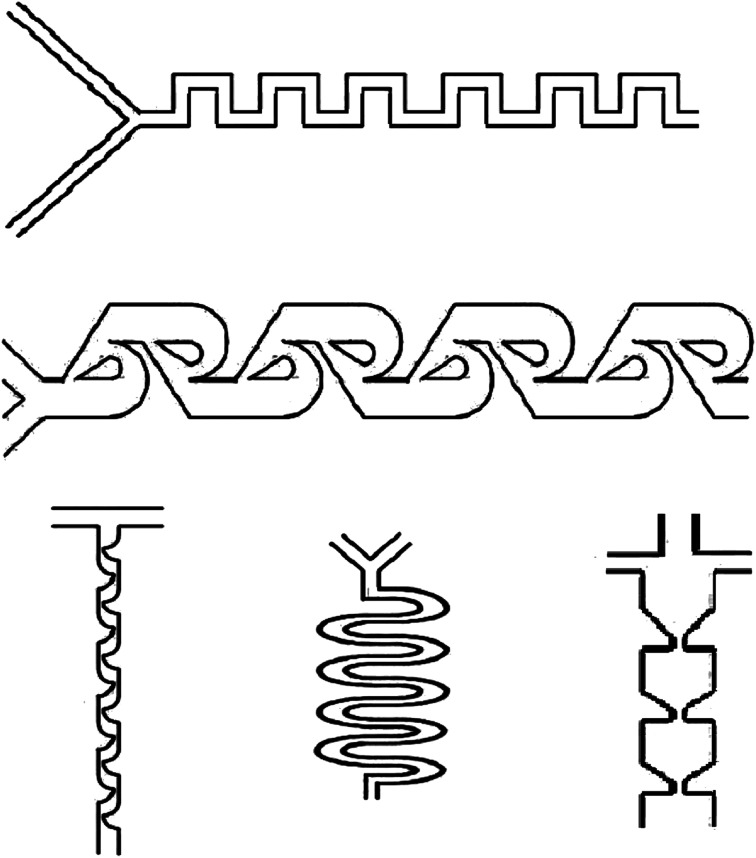
Micromixer microfluidic devices for use in drug delivery.

Microbubbles have various uses, like therapy in acute coronary syndromes, *etc.* Microbubble sizes, the quantity of encapsulated bubbles in a droplet, and the middle layer thickness are managed *via* altering the microfluidic structure, and the phase flow rates. In general, inaccuracy in management impacts the double emulsion morphology, which is usually spherical.^115^ Monodisperse microgels in O/W/O double emulsions are used to arrest the shape. The microgel moves the interior oil droplet to the edge of the water phase shell droplets so that the double emulsion adopts an anisotropic shape.^116^ The parallel microfluidic device constructed for making double emulsions by a single interior core contains two connected flow-focusing junctions, where the second junction channel has a greater diameter and height. The device can make droplets with a diameter variation lower than six percent (Fig. 6).^117^

#### 3.2.2 One-step methods

Like the two-step approach, which utilizes two sequential emulsifications, the single-step approach just utilizes one step to make double emulsions and multiple emulsions. Microcapillary devices, including circular glass capillary channels inserted into a square glass channel to make a co-axial structure, are used in the one-step approach.^118^ The desirable adjustments can be achieved by making the interior square channel dimension and the circular capillary outer diameter equal. The interior and the central fluids are flowed in the same path, into the interior circular capillary channel and the outer co-axial section, whereas the external fluid is injected into the outer co-axial section in the opposite path.^118^ This structure created a co-axial flow, which was broken at the outlet orifice to create double emulsions. The injection capillary possessing two different interior channels enabled the structure of double emulsions by different interior droplets.

Furthermore, double emulsions that include multiple or enormous quantities of interior droplets and multiple sections are produced utilizing multiple bore injection capillaries with different interior tubes to separate each fluid.^119^ Likewise, the co-axial capillary microfluidic devices are utilized for creating monodisperse double emulsions, including an ultra-thin shell that employs a single-step emulsification. The device involves a hydrophobic narrow injection capillary inserted inside of a second square capillary, a little narrow capillary is inside the injection capillary, and an additional circular capillary is inserted in the square capillary at another front to limit the flow adjacent to the injection point.^120^ This approach formed a biphasic flow that allowed one fluid to flow up the capillary channel wall and encircle a second fluid flowing into the capillary channel center.^120,121^

### 3.3 Micromixing in microfluidic devices

Mixing is a commonly practiced unit process in biochemical applications. Hence, microfluidic devices (at Reynolds numbers more than 100) can be utilized for the creation of drug delivery nanoparticles in microchannels.^122^ In macro-fluidic devices, fluid mixing usually depends on convection effects; however, mixing in the micro-fluidic devices is usually performed in the micro tunnels with specific structures to achieve higher surface-to-volume ratio and increase the mass transfer efficiency. Moreover, flow regimes in the microfluidic devices are laminar (Reynolds number less than 1), and flow rates are usually low; as a result, the fluid layers flow in parallel structures without disturbance between the layers, and the fluids mixing is mostly reliant on diffusion with low mixing efficiency.^123,124^ As a result, laminar flow within traditional microchannels avoids turbulence that is present under common conditions. However, micromixers, microfluidic devices with disruptive patterns, have an essential impact on the performance of fluid mixing and can be utilized to mix fluids in microchannels.^125^

**Fig. 7:**
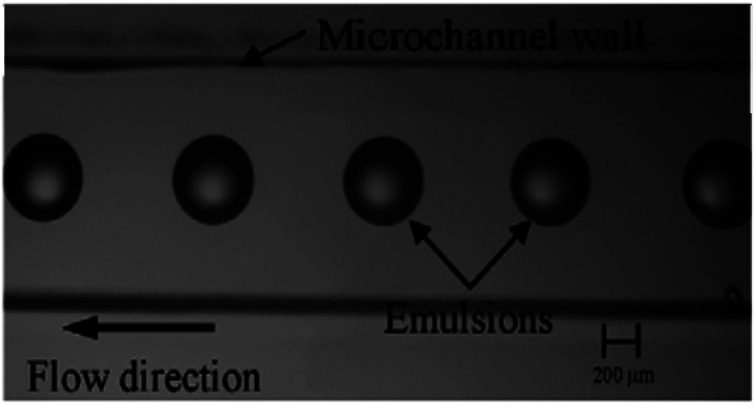
Single emulsion created in microchannels.

Micromixers can be used in stand-alone or one microfluidic systems. Moreover, investigating micromixers is significant in order to comprehend transport phenomena on the microscale. Micromixers, which are utilized for encapsulation in drug delivery, are based on micro-electromechanical system technology, such as PDMS, silicon, and glass. Micromixers are categorized as active or passive due to the mixing system, which passive micromixers, like chaotic advection, serial lamination, and injection, play an essential role in the drug delivery system (Fig. 7).^122^

#### 3.3.1 Active micromixers

Active micromixers, based on various external energy sources in order to disturb the fluids, raise the contact area to improve the mixing effect. Depending on the external energy sources types, active micromixers can be identified as pressure, thermal, electrical, and magnetic field-driven.^126^

#### 3.3.2 Passive micromixers

Passive micromixers are based on the microchannel structures in order to improve the chaotic advection and molecular diffusion for adequate mixing. There are numerous passive micromixers created by scientists and based on the structure, passive micromixers can be classified as chaotic advection, microvortex, *etc.*; based on the structure dimensions, passive micromixers can be sub-categorized as three-dimensional and two-dimensional.^122,127^

Chaotic advection is a method for improving the mixing efficiency by employing geometric patterns to cause transversal flow components, which extend and fold fluid volumes across the microchannel flow area. The chaotic advection mixer uses further microchannel surfaces in order to produce turbulent mixing in a continuous flow. Therefore, the fluid is redistributed across the whole channel cross-section and remarkably decreases the Taylor dispersion.^128^

Microvortex methods are promoted to overcome more moderate productivity because of the wasteful production of unwanted polymer aggregates and deliberate diffusive mixing. However, microvortices work under high pressures and high Reynolds numbers; hence, air bubbles can cause severe problems.^129^

## 5 Detection techniques

The principal parameters investigated in the characterization of nanoparticles are size, shape, size distribution, aggregation degree, *etc.* A variety of techniques have been developed to measure these parameters; however, they can be classified into two main groups, X-ray diffraction and transmission electron microscopy.^155^

X-ray diffraction (XRD) is a technique for the panoramic tensile dispersion that is used to determine the atomic and molecular structure of materials in a wide range of disciplines. In 1912,^156^ this non-destructive method was discovered by von Laue, which can reveal properties, such as network parameters, strain, phase composition, preferential order disorder transformation, thermal transformation, and the grain size of crystals, and obtain structural information from a crystalline sample.^157^ The feature of this technique is that a single-color X-ray on a surface of a particular specimen accelerates the beam in different directions, and then the dispersed beam is collected and stacked into a pattern.

In 1931, Knoll and Ruska developed an electron microscopic transmission technique (TEM). Although, X-ray diffraction shows some structural properties, it does not have the ability to create a nanocrystal image in real space.^158^ TEM can display atomic network images and the chemical information of a single nanocrystal (with a spatial resolution below 1 nm).^159^ The mobile charging device processes data digitally in the data recording system, while the energy of the X-ray and electron spectrometers disperses to measure the chemical composition and electronic structure of the sample.^157^ In TEM, an enlarged image or dispersion (10^2^ and 10^6^ times) is displayed on the screen.^160^ As a result, developing research in cancer, virology, materials science, pollution and semiconductor technology relies heavily on this technique.^161^

## 6 Influencing factors on the distribution and size of core/shell nanoparticles

Critical properties resulting from the synthesis of core–shell particles are the particle distribution and size. The band gap energy between the conduction and valence band decreases with increasing particle size. This is considered a change in the properties of the particles. Additionally, particles in the entire range of 1–100 nm do not display similar properties.^162^ Particles with optical and quantum mechanical properties are only possible below a specific size. Also, the importance of magnetic nanoparticles is highlighted in certain cases, such as the delivery of controlled medication and MRI *in vivo* applications.^163^ Additionally, since particles in the blood tend to coagulate in the cell, particles larger than 50 nm cannot be used for *in vivo* applications. Also, in special cases, large-sized particles cannot be accepted.^164^

Fundamental factors also indicate that smaller particles with a small size distribution have less tendency to agglomerate.

**Fig. 8:**
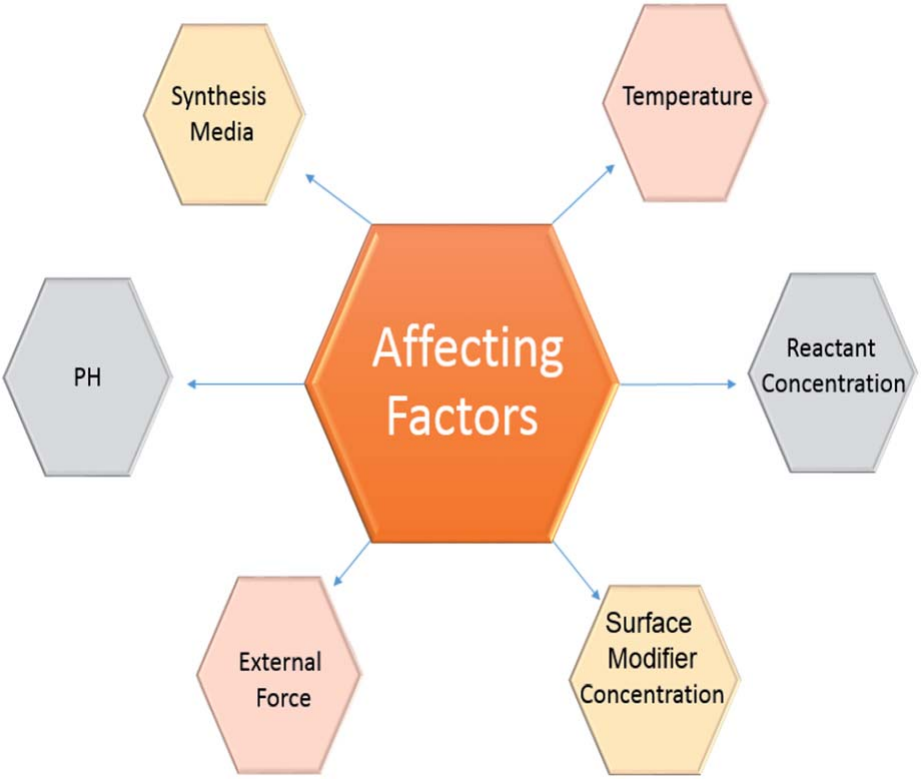
Influencing factors on the distribution and size of core/shell nanoparticles.

Consequently, it is possible to consider the critical factors displayed in Fig. 8.

### 6.1 pH effect

In general, the pH impacts the particle size grown in reaction media. Indeed, due to the fact that OH or H+ ions interact immediately with the reaction, intermediate pH changes are very effective in reducing and depositing reactions.^165^ In the oxidation reaction, the component with the highest potential is decreased by oxidation and reduced in combination with the reduction potential. Therefore, controlling the reaction with pH is very favourable.^166^ The measurement of surface changes is also controlled by the pH adjustment. The surface loss of the hydraulic core depends on pH and the polyelectrolyte modifications are consistent for the surface changes of the shell's coating. As a result, in order to maintain a constant shell coating, the synthetic pH environment is tuned by considering the maximum actuator force, or the difference in bed loading of the magnetic material and the core.^167^

### 6.2 External force effect

Apart from the reaction factors, the use of outside forces in a scheme can be helpful in controlling the particle size. Between the various forces that are used, electrical forces and sonication devices are more commonly applied for particle synthesis.^168^

### 6.3 Temperature effect

Temperature is a well-known reaction parameter utilized to manage the reaction kinetics for nanoparticle synthesis. Generally, for exothermic reactions, the reaction rate declines by increasing the temperature, but the reverse happens in the endothermic reactions. Typically, the temperature is not an essential factor for reduction reactions; however, it is an essential parameter for thermal decomposition, polymerization, or precipitation reactions.^169^ Commonly, a high temperature is needed for thermal decomposition reactions, while exothermic polymerization reactions can take place at lower temperatures. In precipitation reactions, the conditions slightly change. For particles to precipitate, a reaction is classified into these steps: nucleation, growth, and agglomeration. Consequently, particle size depends on the relative rates of these steps.^170,171^

### 6.4 Reactant concentration effect

The concentration of reactants is a necessary parameter to control the shell thickness and core size. The reaction equilibrium that dictates the final particle size takes place in two steps: the reaction among the reactants to create nuclei and contact between the diffusion of molecules to the nuclei surface and displacement to build up to the final particle size.^172^ The initial step is recognized as the reaction step, which is observed by growth. The initial step is fast. As the reaction rate is quick, the final particle size depends on the growth procedure. No particular trend described in the article relates to the change in particle size by increasing reactant concentration. However, the trends in particle size are observed to be dependent on the classes of particles and the synthesis mechanisms.^173^ Generally, an increasing size trend is observed because of increases in the overall amount of the product. When the reactant concentration is greater, the reaction rate can increase, which results in a greater amount of nuclei. This leads to smaller particles. In T-microemulsion methods, by increasing the reactant concentration, the particle size declines due to a change in the media of particle structure.^174^ Increasing the reactant concentration causes the quantity of reacting classes to increase for specific micelles. Consequently, the final particle size declines. The shell structure on a core particle typically proceeds by a heterogeneous nucleation process.^175^ The nuclei of the shell material is stored on the core surface, which is preserved, and expands on the surface, rather than creating new nuclei in the bulk phase. Consequently, a fast reaction rate favours a uniform coating. Hence, a high reactant concentration is more beneficial for core/shell nanoparticle synthesis. Generally, the core/shell particle size or shell thickness increases by enhancing the reactant concentration.^176,177^

### 6.5 Surface modifier effect

The concentration of the surface modifier plays an essential part in the control of the size of core/shell nanoparticles. Ionic surface modifiers diffuse onto the core surface and create a stable particle surface, which results in the formation of more small core particles by reducing the agglomeration of the particles.^178^ As a result, the shell material is required to selectively and consistently transfer onto the core surface. Experimental results exhibit that the materials that form the surface modifiers are responsible for the present surface modification. They diffused onto the surface and controlled the size, and by surface modification, improve the driving force for a shell structure in core/shell synthesis.^179^ The amount of the surface modifier present is controlled by the modifier concentration in solution. It increases with concentration until reaching a specific limit, at which point it plateaus. The final size of the core/shell particles can be controlled by utilizing a surface modifier. However, the particle size distribution mostly depends on the particle surface charge. Generally, a small distribution of particle size can be achieved with ionic surface modifiers.^180^

### 6.6 Synthesis media effect

The synthesis mechanism is a crucial part of managing the final size and distribution of particles. Generally, for a bottom-up method,^16^ a chemical route is used to synthesize the particles. The chemical route is used in both bulk phases and microemulsion methods. Either of these mechanisms has essential advantages and disadvantages, which are neglected depending on the condition.^181^

## 7 Conclusion

Various types of core–shell structures exist, which can be classified based on the presence of metal, non-metal, and polymer components. The core–shell structures maintain stability when re-dispersed in water. Core–shell structures include a unique type of metal-polymer composite, which is utilized in many fields, including biomedicine. The principal focus in research and technology development is the significant improvement to the creation of new types of core–shell nanostructures for modern biomedical applications. In addition, the low-cost, scalability and high-throughput efficiency of microfluidic synthesis strategies have established them as promising technology for the industrial production of pharmaceuticals. Over the past decade, core–shell nanoparticles have been actively involved in various advanced applications, such as drug delivery. Some of the important challenges in this field are ensuring compatibility between the core and shell and using the unique core and shell features individually. In addition, the promotion of both core and shell materials for specific programs, especially in the field of biomedicine, has been the focus of recent research efforts. Briefly, the integration of modern technology in the production of microfluidic devices not only increases production output but also provides an attractive platform for different environmental applications.

This review paper represents the research areas of core–shell nanoparticles that have been expanding in new directions in the past decades. Subsequently, this field will proceed to create processes for modification and synthesis. In this work, we reviewed several papers to provide readers with a collection of core–shell review papers. By reviewing the advantages or disadvantages of the papers, we aim to promote all science as valuable in a respectful manner.

Nanoparticle applications in the biomedical field with the invention of new methods and more compatible core–shell nanoparticles for drug delivery has afforded exciting alternatives to the present methods. The innovation of enhanced microfluidic devices and core–shell nanoparticles will decrease the industrial cost, and dramatically improve the disease treatment efficiency. The appearance of core–shell nanoparticles has affected all the fields in medical and biomedical engineering, while developing and enhancing the already existing methods along with the exploration of new and advanced techniques. The creation of new nanoparticles with enhanced properties has opened the door to utilize them in several fields and to be efficiently employed in real-life situations. Likewise, several drug delivery platforms have been improving with the purpose of targeted drug delivery. However, an excellent method with a selective and sensitive drug delivery system with appropriate core–shell nanoparticles has not yet been invented to be utilized in various systems. As a result, there is a broad future scope for the research to lead this technology for its end-use.

## List of symbols and abbreviations

CSNs: Core–shell nanoparticles
NPs: Nanoparticles
XRD: X-ray diffraction
TEM: Transmission electron microscopy
MSNs: Mesoporous silica-based nanoparticle

## Conflicts of interest

There are no conflicts to declare.

## Supplementary Material
